# The Control of Venereal Disease in Denmark

**Published:** 1913-09-27

**Authors:** 


### THE CONTROL OF VENEREAL DISEASE IN DENMARK.

### Provision for Treatment and Impersonal Notification.

By "THE HOSPITAL'S" SPECIAL COMMISSIONER.

The promised inquiry under Government auspices into the question of the control of venereal disease, and the publicity given to the discussion on this subject at the International Medical Congress, have directed attention to the methods in use in other countries. The one outstanding feature of the discussion was that it was agreed on all sides that the first essential step to take is to provide adequate and easily accessible means of treatment. Before such is available it is impossible to exercise any degree of compulsion on the sufferers from this class of malady. This view is the one emphasised in the recent Report issued by the Local Government Board. The progress made in Denmark in this direction has been referred to, and the writer found it interesting to examine the existing provision for the treatment of venereal disease in Copenhagen. In passing, it may be mentioned that there is a Board of Health in this city presided over by the chief of police, which includes the burgomaster and the chief medical officer, with two members of the City Council. This Board controls the management of all sanitary matters. From the point of view of the sufferers from venereal disease, the important fact is the availability of some nine consultation centres where these patients may go for treatment. These stations are scattered in various parts of the town, and the medical men appointed thereto attend at fixed hours. The names and hours of consultation are given on small posters displayed, among other places, at the kiosks where stamps, papers, and other articles may be purchased. The treatment dispensed at these centres is, of course, gratuitous, and the doctors are paid by the municipality. The appertaining system of impersonal notification will be referred to later.

### The Consultation Centres.

This first stage provision is, then, on a fairly extensive scale considering the size of the town. The population of Copenhagen may be taken as half a million, so that a proportional provision for London in this respect would entail the establishment of nearly one hundred consultation centres. In Copenhagen at the present time about 36 per cent. of the new cases of venereal disease seek advice at these centres. Naturally, the general hospitals and the skin hospitals of the town treat a certain number of these cases in their out-patient departments, as in London. But when the extent of the provision for in-patient treatment is considered, Copenhagen will be found to be much better off in this respect than London. As was pointed out in the Local Government Board Report by Dr. Johnstone, London is very poorly equipped with beds for the treatment of venereal disease, there being practically no beds reserved for these cases in the general hospitals, while the Lock Hospital in the Harrow Road only provides 120 female beds and twenty-one cots, and the male hospital will in the near future accommodate about thirty in-patients.

The treatment and accommodation of venereal patients in Poor-Law institutions in London leaves much to be desired. In Copenhagen there are available close upon 400 beds for cases of venereal disease. In the Rudolf Bergs Hospital there are about 165 beds for this class of disease, the remainder being devoted to skin diseases. In the magnificent and new Rigs Hospital, in the Kommune Hospital, in the Blegdaurs Hospital for Infectious Diseases, and in some of the other hospitals, there are wards reserved for venereal disease, affording a total accommodation of over 360 beds for a population one-tenth that of London.

### The Impersonal Notification System.

Turning now to the question of administrative control, it must first be recorded that in 1906 State "regulation" was given up as a failure in Copenhagen. Experience showed there, as elsewhere, that the professional prostitute is not the principal agent in the spread of venereal disease. Together with the institution of the above-described consultation centres was adopted a system of impersonal notification.

Corresponding to our notification forms for ordinary infectious diseases the Danish doctors have to fill up a return, which is sent in weekly, though, of course, means are available for the immediate admission into hospital of cases of scarlet fever and such like. This weekly return consists of a form containing spaces for twenty diseases and their classified age and sex groups. It may be mentioned that on the list, in addition to those diseases notified here, are such diseases as pneumonia, rheumatic fever, influenza, and tonsillitis. On the same form there are seven other spaces allotted respectively to gonorrhœa, soft sore, acquired coitional syphilis, syphilis insontium, congenital syphilis, scabies, and delirium tremens. Against these last seven the doctor is not required to add the names and addresses of the patients he has seen, whereas in the other cases this has to be done.

### Professional Secrecy and Control.

A patient suffering from venereal disease, when he attends a doctor, gives his name and address as a matter of course, but so long as he remains under treatment and is satisfactorily following the treatment, no use is made of this information, which is regarded as confidential. Should, however, the patient neglect to attend until he is cured or harmless, then the doctor sends to his address a sort of warning notice requiring his further attendance. If no notice is taken of this, and the doctor is of opinion that the case is still in a state dangerous to the community, the police are notified, and the case is followed up by them. Similar measures are adopted in the case of hospital patients; those who are in-patients are usually kept under supervision in the wards until it is safe to let them out, but some cases are allowed to "report" themselves under proper medical care. In actual practice the police have few cases handed over to them, and among the better-class private patients there is little or no official interference employed. Pamphlets couched in plain language setting forth the dangers of the disease are distributed to all patients coming under treatment.

Immediately after 1906 there was, as might be expected, a noticeable increase in the number of cases of venereal disease reported, but last year some decrease was recorded. The curves for the three classes—gonorrhœa, soft sore, and syphilis—are all higher in the last quinquennium, 1908-1912, than in the previous one, 1903-1907. But the general opinion seems to be that in the future a lower level will be maintained.

